# T Cells in Autoimmunity-Associated Cardiovascular Diseases

**DOI:** 10.3389/fimmu.2020.588776

**Published:** 2020-10-07

**Authors:** Daniella Muallem Schwartz, Aarohan M. Burma, Moses M. Kitakule, Yiming Luo, Nehal N. Mehta

**Affiliations:** ^1^ Laboratory of Allergic Diseases, National Institute of Allergy and Infectious Diseases, National Institutes of Health, Bethesda, MD, United States; ^2^ Rheumatology Fellowship Program, National Institute of Arthritis, Musculoskeletal, and Skin Diseases, National Institutes of Health, Bethesda, MD, United States; ^3^ Section of Inflammation and Cardiometabolic Diseases, National Heart, Lung, and Blood Institute, National Institutes of Health, Bethesda, MD, United States

**Keywords:** T cells, autoimmunity, cardiovascular, systemic lupus erythematosus (SLE), rheumatoid arthritis (RA), psoriasis, vasculitis

## Abstract

T cells are indisputably critical mediators of atherosclerotic cardiovascular disease (CVD), where they secrete pro-inflammatory cytokines that promote vascular pathology. Equally well-established is the fact that autoimmune diseases, which are mediated by autoreactive T cells, substantially increase the risk of developing CVD. Indeed, as immunomodulatory treatments have become more effective at treating end-organ pathology, CVD has become a leading cause of death in patients with autoimmune diseases. Despite this, investigators have only recently begun to probe the mechanisms by which autoreactive T cells promote CVD in the context of autoimmune diseases. T cells are best-studied in the pathogenesis of systemic vasculitides, where they react to self-antigen in the vessel wall. However, newer studies indicate that T cells also contribute to the increased CVD risk associated with lupus and rheumatoid arthritis. Given the central role of T-cell-derived cytokines in the pathogenesis of psoriasis, the role of these factors in psoriatic CVD is also under investigation. In the future, T cells are likely to represent major targets for the prevention and treatment of CVD in patients with autoimmune diseases.

## Introduction

Atherosclerotic cardiovascular disease (CVD) is one of the leading causes of morbidity and mortality in the United States and globally (1, 2). Over the last several decades, inflammation has emerged as a key driver of atherosclerotic CVD, as well as a major therapeutic target (3, 4). In particular, a large body of preclinical and clinical studies implicate CD4+ and CD8+ T cells in the pathogenesis of atherosclerotic CVD (2). T cells are enriched in atherosclerotic plaque, where they recognize lipid- and endothelial-derived antigenic peptides and secrete proinflammatory cytokines (2, 5–7). Moreover, adoptive transfer of effector T cells promotes atherogenesis in murine models, whereas transfer of regulatory T (Treg) cells is protective (8–10). Taken together, these and other studies clearly establish that T cell-mediated immunity is a major modulatory of atherosclerotic CVD pathogenesis (2, 11–13).

Systemic autoimmune diseases are characterized by aberrant adaptive immune responses to autoantigens. Autoreactive T cells play a central role in the pathogenesis of autoimmunity. Severe early-onset autoimmunity is a prominent feature of immune dysregulation syndromes caused by mutations in T cell specific genes such as *FOXP3* and *CTLA4*. Moreover, common autoimmune diseases are strongly associated with polymorphisms in genes that are preferentially expressed in T cells (14). A large body of human and murine studies has established multiple mechanisms by which T cell dysfunction promotes systemic autoimmunity in a variety of common rheumatic diseases including rheumatoid arthritis (RA), systemic lupus erythematosus (SLE), myositis, psoriasis/psoriatic arthritis, and vasculitis (15).

Considering the major pathogenic role of T cells in both atherosclerosis and systemic autoimmunity, it is perhaps unsurprising that autoimmune diseases represent a major risk factor for CVD (15, 16). Furthermore, CV risk is reduced in patients with rheumatic diseases who achieve clinical remission. This observation has led to multiple studies testing the efficacy of anti-inflammatory therapies as a primary prevention strategy for CVD in patients with autoimmune disease (17–21). In order to select the most promising therapeutic targets, it is critical to understand the specific mechanisms by which T cells interact with other dysregulated populations to promote CVD in patients with autoimmunity. This review will focus on the mechanistic evidence implicating T cells as drivers of vascular inflammation, starting with primary vasculitides and then focusing on three prototypic systemic autoimmune diseases: RA, SLE, and psoriasis. We will also briefly review the efficacy of T-cell-directed therapies in the treatment of autoimmunity-associated vascular dysfunction.

## Overview of T Cells in Primary Vasculitides

A review of T-cell mediated inflammation in autoimmunity-associated CVD would be incomplete without a discussion of T cells in the context of primary vasculitides. Vasculitides are a group of heterogenous disorders classified according the size of the vessel they predominantly affect: small-, medium-, and large vessel (22). Vasculitis can develop as secondary to various underlying medical conditions or constitute a primary autoimmune disease, where the vasculature is the target of immune-mediated pathology. The etiology and pathogenesis of primary vasculitis are not completely understood, but accumulating evidence has suggested a pathogenic role for T cells. This role has been most extensively explored in two prototypical vasculitic disorders that will be the focus of this review: the small-vessel disease antineutrophil cytoplasmic antibody (ANCA)–associated vasculitis (AAV) and the large-vessel vasculitis giant cell arteritis (GCA).

## T Cells in ANCA-Associated Vasculitis

**Figure 1:**
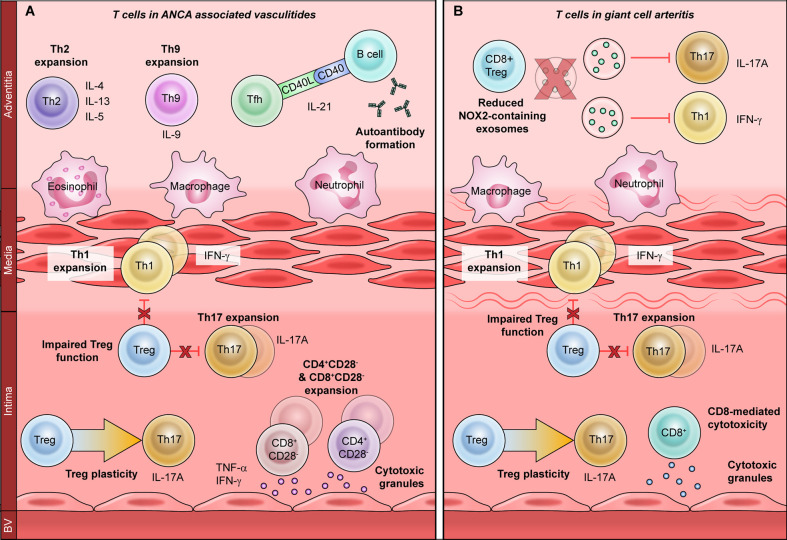
The role of T cells in primary vasculitides. T cells promote vascular inflammation in primary vasculitides through a variety of mechanisms. Expansion of proinflammatory T helper (Th)-1 and Th17 subsets is associated with both ANCA-associated vasculitis **(A)** and giant cell arteritis **(B)**. Regulatory T cells are also hypofunctional in both vasculitides and display increased plasticity, or conversion to Th17 cells. Th17 cells recruit neutrophils and macrophages to promote acute vascular inflammation, whereas Th1 cells regulate macrophages to promote chronic damage. In ANCA-associated vasculitis **(A)**, T follicular helper (Tfh) cells promote the production of anti-neutrophil cytoplasmic antibodies (ANCA), which induce vascular inflammation. Th2 and Th9 cells produce IL-4, IL-13, IL-5, and IL-9, which promote eosinophilic vascular inflammation. CD4+CD28− and CD8+CD28− cells produce atherogenic cytokines such as TNF-α and IFN-γ, as well as directly damaging the vasculature by releasing cytotoxic molecules. Mechanisms of T cell dysfunction specific to giant cell arteritis **(B)** include enhanced CD8-mediated cytotoxicity and reduced anti-inflammatory function of CD8+ Tregs. This is due to reduced production of NOX2-containing exosomes, which inhibit the proliferation of Th1 and Th17 cells.

The AAV comprise three clinical syndromes: granulomatosis with polyangiitis (GPA), microscopic polyangiitis (MPA) and eosinophilic granulomatosis with polyangiitis (EGPA). Because T cells are critical orchestrators of antigen-specific autoimmunity, T cell dysfunction in the context of AAV is thought to directly promote disease (**Figure 1A**) (23). CD4+ T cells are considered particularly important to disease pathogenesis, since effector memory CD4+ T (T_EM_) cells are persistently expanded in AAV (24, 25). Indeed, T_EM_ cells migrate from the peripheral circulation into inflamed tissues during recurrent disease, indicating that they may drive disease relapse (26–28). Moreover, AAV-associated T_EM_ cells express natural killer group 2D (NKG2D) receptor, giving them the capacity to mediate vascular injury through cytotoxicity (29, 30). Taken together, this suggests a central role for CD4+ T cells in AAV-associated vascular inflammation. As in atherosclerotic CVD, CD4+ T cell dysfunction AAV can occur through three broad mechanisms: dysregulated T helper (Th) differentiation, CD4^+^CD28− T cell expansion, and impaired regulatory T cell (Treg) function.

CD4+ T cells differentiate into various effector subsets (Th1, Th2, Th17, Th9, Th22, T follicular helper or Tfh), each of which mediates a discrete immunological response through the secretion of subset-specific effector cytokines (31). Studies have revealed a shift toward Th2 response in patients with generalized GPA with systemic vasculitis, whereas a Th1 response is seen in localized GPA with predominantly nasal lesions (32, 33). GPA is also associated with Tfh expansion, which may contribute to ANCA autoantibody production, whereas Th2 and Th17 expansion have been observed in EGPA (34–37). Proteinase-3 (PR3), the key pathogenic antigen associated with GPA, can itself modulate Th differentiation: PR3-expressing apoptotic cells promote a Th2/Th9 response, while PR3-ANCA promotes Th17 differentiation (38).

Expansion of the proinflammatory and cytotoxic CD4^+^CD28− T cell subset has been consistently reported in GPA (39–43). CD4+CD28− T cell expansion is associated with latent cytomegalovirus (CMV) infection and confers a poor prognosis (39, 40). However, it is not yet apparent whether CD4+CD28− T cells contribute to AAV-associated vascular inflammation, or whether they worsen outcomes through other mechanisms (41, 44). For example, CD4+CD28− T cell expansion is associated with impaired immunological responses to vaccination, which could increase infection-related morbidity (39, 40).

In contrast to T effector cells, Tregs are key negative regulators of inflammation that promote immune tolerance (45). Several studies have described reduced Treg frequency in AAV, but others have reported increased numbers, possibly due to the different methodologies of identifying human Tregs (36, 46–50). Moreover, functional Treg impairment is seen in active AAV and improves during disease remission (46–50). Treg impairment may arise from utilization of a hypofunctional isoform of the Treg-associated master transcription factor Forkhead box P3 (FoxP3), or from enhanced conversion into pathogenic Th17 effector cells (47, 51).

A limited body of data suggests that CD8+ T cells may also play a role in AAV. CD8+ T cells promote glomerular injury in murine MPA, and circulating CD8+CD28− T cells are expanded in GPA (52, 53). A subset of circulating T cells expressing both CD4 and CD8 has also been described in the context of human disease, although the function of this subset is incompletely characterized (54). CD4+CD8+ double-positive T cells are expanded in GPA and exhibit a memory phenotype, with co-expression of CD28 and NKG2D (53). Future investigations will be needed to define the role CD4+CD8+ double-positive cells in the pathogenesis of AAV.

## T Cells in Giant Cell Arteritis

GCA is a large-vessel vasculitis of unknown etiology that occurs mainly in individuals over age 50 (55). The pathological hallmark of GCA is granulomatous arterial wall inflammation, with infiltration of T lymphocytes, macrophages, dendritic cells (DCs) and multinucleated giant cells (56). While the pathogenesis of GCA is incompletely understood, over two decades of work implicate CD4+ T helper cells as major drivers of the pathological immune response (**Figure 1B**) (57–60).

GCA patients have marked expansions of Th1 and Th17 cells, which are thought to differentiate from a common precursor but promote two discrete pathologies (60, 61). Th17 cells promote neutrophil and macrophage recruitment, and Th17 expansion correlates strongly with signs of active inflammation. Th17 expansion also normalizes promptly with corticosteroid treatment, implying that Th17 cells primarily induce acute vessel inflammation (60). Conversely, Th1 expansion is associated with chronic persistent inflammation and vascular remodeling (60, 62). The Th1 effector cytokine IFN-γ activates macrophages and promotes giant cell formation (60). IFN-γ-stimulated macrophages also secrete platelet-derived growth factor (PDGF) and vascular endothelial growth factor (VEGF), which induce vascular hyperplasia and neoangiogenesis, ultimately causing luminal occlusion and ischemia (63–65). Notably, the Th1 responses in GCA are resistant to corticosteroid treatment which may explain why even patients in remission are at a high risk of subsequent vascular events (62, 66).

Reduced Treg frequency and Treg dysfunction have also been reported in GCA, though these findings are complicated by the different methodologies used to identify human Tregs in various studies (62, 67). As in AAV, Tregs derived from GCA patients have impaired suppressive ability and utilize the hypofunctional FoxP3 isoform (68). Treg plasticity has also been implicated in GCA pathogenesis, as FoxP3+T cells expressing the Th17-associated cytokine IL-17A have been identified in temporal artery biopsies (69, 70). Unexpectedly, temporal artery expression of IL-17A is associated with a favorable prognosis, indicating that IL-17A+ Tregs may retain at least some suppressive capacity (71).

Although the role of CD8+ T cells in large vessel vasculitis is less clearly defined, CD8+ dysfunction and CD8+-specific transcriptomic changes have been reported in association with GCA (72, 73). CD8+ cells can also function as regulatory cells and promote immune tolerance, like their CD4+ counterparts (74). CD8+ Treg function is impaired in elderly individuals, with the highest degree of impairment seen in elderly individuals with GCA (75). This is thought to result from reduced production of NADPH oxidase 2 (NOX2), which CD8+ Tregs release in exosomes to dampen CD4+ proliferation and resultant autoimmunity (75).

## T Cells in CVD Associated With Rheumatoid Arthritis (RA)

**Figure 2:**
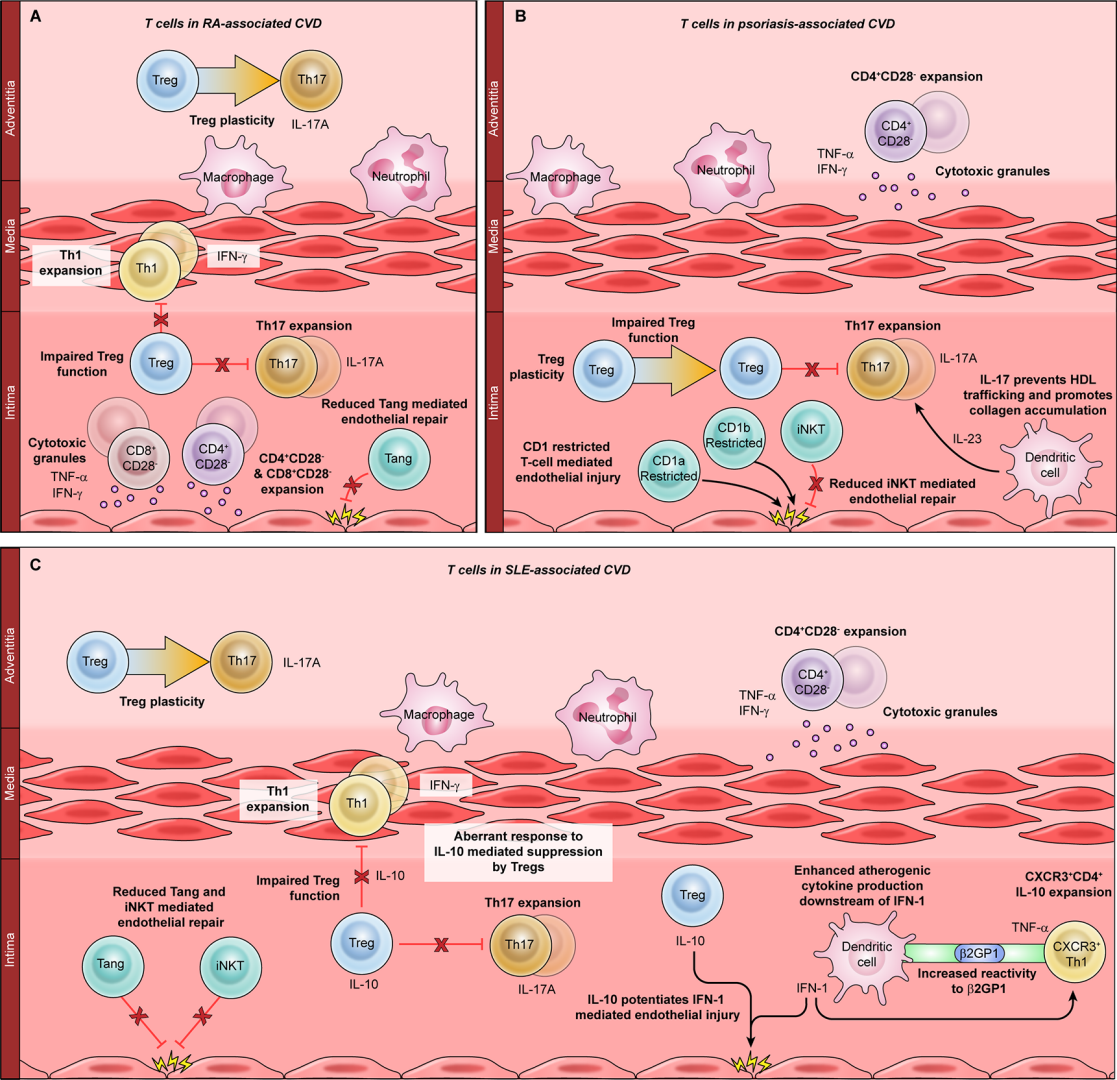
The role of T cells in autoimmunity-associated cardiovascular disease (CVD). T cell dysfunction has been implicated in CVD associated with rheumatoid arthritis (RA, **A**), psoriasis **(B)**, and systemic lupus erythematosus (SLE, **C**). Mechanisms common to all three autoimmune diseases include expansion of CD4+CD28− cells, which produce atherogenic cytokines such as TNF-α and IFN-γ, and release cytotoxic molecules that damage the vasculature. Proinflammatory T helper (Th)-1 cells are expanded in RA **(A)** and in SLE **(C)**; Th1-mediated atherogenesis is enhanced by the SLE-associated cytokine IFN-1. Th17 cells are expanded in all three autoimmune diseases and are particularly important for psoriatic CVD. Th17 differentiation is enhanced by the psoriasis-associated cytokine IL-23 and inhibited by regulatory T cells (Tregs). Treg dysfunction and plasticity, or conversion to Th17 cells, are implicated in CVD associated with RA, SLE, and psoriasis. In SLE, the Treg-derived cytokine IL-10 synergizes with the dendritic cell-derived cytokine IFN-1 to promote atherogenesis. Angiogenic T cells and CD1-restricted T cells such as invariant natural killer T (iNKT) cells can directly mediate endothelial damage and repair. Dysfunction of these subsets is seen in RA, SLE, and psoriasis.

Rheumatoid arthritis (RA) is an autoimmune disease with a United States prevalence of 0.5 to 1 (76). Although joint destruction is the hallmark of RA, almost 50% of patients develop devastating extra-articular manifestations, including CVD (77). The association between RA and CVD is extremely well-established, with multiple studies demonstrating a 1.5-fold increased risk of CVD in RA patients (17, 78, 79). Traditional CV risk factors clearly contribute to CVD in RA patients, including hyperlipidemia, obesity, and smoking. However, traditional CV risk factors do not fully account for the increased CVD risk burden in RA, RA disease severity correlates with CVD, and immunomodulatory treatments reduce the risk of CVD in RA patients (17, 78, 79). Observational studies suggest that abatacept, a T cell immunomodulator, is more effective at preventing CVD in RA patients than TNF inhibitors, which act on multiple immune cell populations (17). Taken together, these data strongly implicate primary immune dysregulation, including T cell dysfunction, as a central driver of CVD in RA patients (**Figure 2A**).

T cells are central drivers of RA disease pathogenesis, promoting joint destruction through various mechanisms including secretion of proinflammatory cytokines, B cell activation, regulatory T cell dysfunction, and direct cytotoxicity – many of the same mechanisms implicated in CVD pathogenesis (2, 80, 81). Terminally differentiated T_EM_ CD4+ and CD8+ T cells are expanded and correlate significantly with coronary artery calcifications in RA patients, suggesting a pathogenic role (82). RA is also characterized by CD4+CD28− cell expansion, which is closely tied to the development of atherosclerotic CVD (83, 84). Accordingly, the frequency of circulating CD4+CD28− cells significantly correlates with preclinical atherosclerosis in RA patients, indicating that these cells may be major inducers of RA-associated CVD (84, 85). CD8+CD28− cells have also been described in association with RA-associated CVD, although the role of this subset is not as clearly defined (86).

Like primary vasculitides, RA is characterized by expanded proinflammatory Th1 and Th17 cells (81). In murine autoimmune arthritis models, pathogenic Th17 cells interact with vascular endothelial cells to promote both angiogenesis and joint destruction through production of placental growth factor, which correlates with IL-17A levels in RA patients (87). This provides strong mechanistic evidence that Th17 cells can promote RA-associated vascular injury. A subset of angiogenic T cells, characterized by coexpression of CD3/CD31/CXCR4, can also attenuate vascular injury by promoting endothelial repair (88). Two studies have analyzed the frequency of angiogenic T cells in RA patients, with discrepant results (36, 88). This could be related to differences in patient populations: one study focused on European patients with a high risk of CVD whereas the other investigated Asian patients with very few CV risk factors. This would be consistent with prior observations that RA-associated CVD is driven by complex interactions between traditional CV risk factors and systemic inflammatory mediators (89).

## T Cells in CVD Associated With Psoriasis

Psoriasis is a T-cell-mediated autoimmune disease whose hallmark symptom is chronic skin inflammation. Psoriasis has a prevalence of 2% to 3% and causes extracutaneous disease in up to 30% of patients (90). Psoriasis is associated with a number of comorbid conditions that increase the risk of atherosclerotic CVD, including metabolic syndrome and chronic kidney disease. As in other autoimmune conditions, CVD risk in psoriasis patients correlates with disease severity and improves with immunomodulatory therapy (91, 92). An extensive body of work over the last several decades has shown that psoriasis is a T-cell-mediated disease, with Th17 cells emerging as the central drivers of cutaneous pathology (90). Accordingly, blockade of Th17-derived IL-17A and the Th17-inducing cytokine IL-23A are both highly efficacious for skin disease in most patients with psoriasis (93).

Given the centrality of Th17 cells to both atherosclerotic CVD and psoriatic skin disease, it is reasonable to conclude that Th17 cells link psoriatic immunopathology and inflammatory CVD (**Figure 2B**). Accordingly, Th17 cells from murine psoriatic skin lesions migrate to the arterial wall, where they promote atherogenesis by regulating high density lipoprotein (HDL) trafficking and collagen accumulation (94). Moreover, blocking IL-17A and IL-23 prevented psoriasis-related thrombosis in preclinical studies (95, 96). Subsequently, a number of late phase clinical trials tested the effects of blocking IL-17A and IL-23 on aortic vascular inflammation in patients with psoriasis. Although the immunomodulatory treatments caused transient improvements in inflammation, these changes were not sustained (20, 21, 92). This may be due to the role of other T helper subsets in psoriatic CVD, or because these large studies evaluated aortic inflammation instead of a more sensitive primary outcome measure such as coronary artery plaque burden. Indeed, more recent data has shown that biologic therapy reduces coronary plaque and coronary inflammation over a 1-year period of treatment (18, 97). Additional studies are ongoing that will use a variety of outcome measures, including aortic inflammation and carotid artery pulse wave velocity (NCT02144857, NCT03478280).

Due to the prominent role of Th17 cells in psoriasis, most mechanistic studies of psoriatic CVD have focused on the Th17 lineage and its associated cytokines. However, other T cell subsets have also emerged as potential modulators of atherogenesis in patients with psoriasis (**Figure 2B**). As for many other immunological disorders, several of these studies have focused on the role of CD4+CD28− cells. Circulating and skin-resident CD4+CD28− cells have been identified in patients with psoriasis, but their functions have not yet been defined in this population (98, 99). iNKT cells, which respond to lipids presented by the CD1d family of antigen-presenting molecules, have also been identified in psoriatic skin (100). In addition to iNKT cells, CD1-restricted cells comprise multiple other subtypes with various specialized immunological functions (101). Autoreactive CD1a-restricted T cells recognize lipid autoantigens in patients with psoriasis, providing a potential link between skin inflammation and CVD (102). CD1b-autoreactive cells promote murine psoriatic skin inflammation but have not been found to induce atherogenesis (103). As more information emerges about the roles of CD1-restricted T cells in human immunity, these cells may emerge as major links between cutaneous disease and atherogenesis in patients with psoriasis.

## T Cells in CVD Associated With Systemic Lupus Erythematosus (SLE)

Systemic lupus erythematosus (SLE) is a chronic systemic autoimmune disease with a prevalence of 30 to 50 per 100,000 (104). SLE is typified by a combination of innate and adaptive immune dysregulation, which act in concert to promote disease pathogenesis (104). T cells have an essential role in SLE pathogenesis, with T effectors directly promoting SLE and Tregs attenuating end-organ pathology (105–107). Like RA, SLE is associated with a significantly increased risk of CVD not entirely explained by traditional risk factors (104).

An emerging body of evidence implicates T cell dysfunction as a key cause of atherogenesis in patients with SLE (**Figure 2C**). Aberrant T cell activation is a prominent feature of SLE-associated CVD, and adoptively transferred CD4+ T cells are sufficient to induce murine SLE-associated atherogenesis (108, 109). Additionally, T cell immunomodulation is an effective therapeutic strategy for CVD in SLE models and is even superior to lipid lowering therapy (110, 111). Atherogenic CD4+ T cells that express the Th1 marker CXCR3 are expanded in SLE, where they migrate to the arterial wall and directly induce vascular pathology. This process is enhanced by Type 1 interferon (IFN-I) signaling, which is a hallmark feature of immune dysregulation in SLE (112). IFN-I derives primarily from plasmacytoid dendritic cells, demonstrating that innate immune dysregulation and T-cell-driven atherogenesis are closely linked in patients with SLE (112). T cells from SLE patients also display enhanced reactivity to plasma β2 glycoprotein I, leading to immune-mediated hypercoagulability, endothelial cell dysfunction, and subclinical atherosclerosis (113).

As in other autoimmune diseases, both Treg dysfunction and abnormal T effector differentiation have been implicated in SLE-associated CVD. Th17 expansion correlates with both disease activity and atherosclerosis in SLE, whereas Tregs are reduced in SLE-associated CVD (108, 114, 115). In murine SLE-associated atherogenesis, pathogenic T effector cells are also resistant to Treg suppression, possibly due to reduced expression of IL-10 receptor (108). IL-10 is a Treg-derived cytokine with anti-inflammatory properties that suppresses T cell proliferation. Intriguingly, IL-10 is elevated in SLE patients, and IL-10 potentiates IFN-I-induced endothelial dysfunction (116). This suggests another link between CD4+ T cell dysfunction, innate immune dysregulation, and atherogenesis. SLE is also characterized by development of high titer autoantibodies, a process mediated by autoreactive B cells and Tfh cells (104). Atherogenesis promotes the differentiation of Tfh cells in lupus-prone mice, augmenting systemic autoimmunity and providing another link between SLE disease activity and atherogenesis (117).

A limited body of data also suggests a role for other T cell subsets in SLE-related CVD. CD4+CD28− T cells are expanded in SLE, but their relationship to atherogenesis is not well defined (118). Angiogenic T cells have also been described in the context of SLE; in contrast to RA, SLE is typified by expansion of angiogenic CD8+ cells but not angiogenic CD4+ cells. However, angiogenic CD8+ T cells do not correlate with SLE-related disease activity, and their role in CVD is indeterminate (119). Invariant natural killer T (iNKT) cells are an innate-like subset of T cells that can rapidly produce proinflammatory or anti-inflammatory cytokines in response to lipid antigens. In SLE patients, iNKT cells with an anti-inflammatory phenotype are atheroprotective, and their loss confers an increased risk of CV events (120). Future studies will be needed to dissect the roles of these and other non-CD4+ T cell subsets in the pathogenesis of autoimmunity-related CVD.

## Common and Disease-Specific Mechanisms of Autoimmunity-Related CVD

While this review has focused on a selected group of representative systemic autoimmune diseases, the risk of CVD is elevated in multiple organ-specific and systemic autoimmune disorders (121–123). It is impossible to comprehensively address every study linking autoimmunity to the development of CVD, but many of the mechanisms implicated are the same ones identified for vasculitis, RA, SLE, and psoriasis. This is perhaps unsurprising, as many genetic variants that predispose individuals to autoimmunity are shared between multiple autoimmune diseases, including polymorphisms in genes critical for T cell differentiation and function, like *HLA-DRB1*, *PTPN22*, and *CD25* (14). Common T-cell-dependent mechanisms of autoimmunity-associated CVD include CD4+CD28− expansion, CD8+CD28− expansion, Treg dysfunction, and proinflammatory cytokine production by T effector cells (Th1, Th17). By contrast, several T cell subsets are thought to promote CVD in the context of specific autoimmune diseases, including angiogenic T cells (SLE, RA), iNKT cells (psoriasis, SLE), and Tfh cells (AAV, SLE). However, it is important to acknowledge that many T-cell-dependent mechanisms have not yet been studied across multiple autoimmune conditions and could be more broadly shared. For example, IFN-1 is best studied in the context of SLE. Accordingly, IFN-1 is described to enhance Th1-mediated vascular damage in SLE but not in other diseases (112). However, IFN-1 is also implicated in the pathogenesis of RA and psoriasis (80, 124); therefore, IFN-1- may enhance T cell-mediated CVD in RA and psoriasis. Similarly, direct immune-mediated destruction of the vasculature is the hallmark of the primary vasculitides but can also be seen in secondary vasculitides related to underlying SLE or RA. Further investigations are needed to differentiate common and disease-specific T-cell-dependent mechanisms underlying CVD in various autoimmune conditions.

## Therapeutic Modulation of T Cells in Autoimmunity-Related CVD

Although T cells are clearly central to the pathogenesis of autoimmunity-related CVD, other cell types also play a major pathogenic role. These include dendritic cells, B cells, monocytes, neutrophils, and platelets (80, 125, 126). Of note, many of these cells directly interact with T cells to promote autoreactivity or induce endothelial injury downstream of T cell dysfunction. Thus, various proinflammatory cytokines and factors can be targeted both to directly repress dysfunctional T cells and to prevent crosstalk between T cells and other critical effectors. Most conventional disease-modifying antirheumatic drugs (DMARDs) modulate the function of multiple immune cell subsets, including T cells. Methotrexate, which improves CVD in RA, psoriasis, and vasculitis, inhibits T cell activation and promotes Treg differentiation (127, 128). Calcineurin inhibitors, which potently block T-cell-receptor signaling, reduce markers of atherosclerotic CVD in SLE (129, 130). Mycophenolate mofetil also represses dysfunctional T cells and has attenuated CVD in murine models of SLE-related atherogenesis (111). Hydroxychloroquine, which reduces subclinical atherosclerosis in SLE, inhibits T cells by blocking the AP-1 transcription factor downstream of T cell receptor activation (131, 132).

T cells can also be efficiently targeted using biological and targeted synthetic DMARDs. Tumor necrosis factor (TNF) inhibitors, IL-6 receptor inhibitors, and JAK inhibitors all inhibit multiple immune subsets, including pathogenic T cells; these agents are all associated with reduced markers of CVD in patients with systemic autoimmunity (80, 126, 128, 133, 134). Biological DMARDs can also block T-cell-derived factors: as noted previously, blockade of Th17-derived IL-17A may ameliorate CVD in psoriasis, although further studies are needed (18, 19, 21, 97). Finally, the biological DMARD abatacept, which is FDA-approved for RA and psoriatic arthritis, directly targets T cell activation by blocking costimulation. Abatacept lowers the frequency of CD28− T cells and reduces CVD risk in RA, with a larger effect than TNF inhibitors and B-cell-directed therapies (135–140). Abatacept did not prove effective in clinical trials for SLE (141); therefore its effects on SLE-associated CVD is unknown. Early-phase clinical trials suggest that abatacept may also be efficacious for LVV and AAV (142, 143), with phase 3 trials ongoing (NCT02108860, NCT04474847). Taken together, these studies demonstrate that targeting dysfunctional T cells is a safe and effective therapeutic strategy for the prevention and treatment of autoimmunity-related CVD and vascular inflammation.

## Generalizability to Atherosclerotic CVD in Patients Without Systemic Autoimmunity

In addition to their role in autoimmunity-related CVD, T cells have an indisputable role in the pathogenesis of atherosclerotic CVD in patients without underlying autoimmunity. Although the focus of this review does not concern T-cell-dependent CVD in the general population, it is worth noting that many mechanisms implicated in autoimmunity-related CVD also promote atherogenesis in the general population. These include Treg dysfunction/instability, production of proatherogenic cytokines by effector T cells, and T-cell-mediated cytotoxicity (2). The presence of these shared mechanisms suggests that therapies efficacious for autoimmunity-related CVD might also be used to treat patients with atherosclerotic CVD. Indeed, T cell modulation with mycophenolate mofetil may be beneficial in atherosclerotic CVD (144); and clinical trials are ongoing or planned for hydroxychloroquine (NCT02648464, NCT04161339, NCT03636152), temsirolimus (NCT03942601, NCT04433572), tocilizumab (NCT03004703), and abatacept (NCT04344873). However, it must also be recognized that patients with systemic autoimmunity develop more inflammation and T cell autoreactivity than patients with atherosclerosis (125). Accordingly, some disease modifying antirheumatic drugs, such as methotrexate, prevent CVD in patients with systemic autoimmunity but not in patients with atherosclerosis (128, 145). Another T-cell-directed strategy involves the use of tolerogenic vaccinations or low-dose IL-2 to induce atheroprotective Tregs (2). Early phase clinical trials are underway to evaluate the potential efficacy of these strategies for CVD in the general population (NCT01284582, NCT03113773, NCT03042741, NCT02508896) but thus far these methods remain untested. Future studies are warranted to determine the generalizability of T-cell-mediated mechanisms of autoimmunity-related CVD to the general population, and the efficacy of T cell immunomodulation for CVD in patients without underlying autoimmunity.

## Conclusions and Future Directions

Over the last several decades, T cells have emerged as major mediators of atherosclerotic cardiovascular disease. The centrality of T cell dysfunction to human autoimmune diseases, and the increased risk of CVD in patients with autoimmunity, has sparked intense interest in the role of T cell dysfunction in autoimmunity-related vascular inflammation. A large body of evidence has established that T cells are central mediators of vascular inflammation in patients with systemic autoimmune diseases, suggesting that they underlie the increased risk of CVD associated with these disorders.

Several broad mechanisms of T cell dysfunction promote autoimmunity-associated CVD. Aberrant T helper differentiation leads to expansion of Th1 and Th17 cells, which migrate to the arterial wall and promote atherogenesis. This proinflammatory cytokine secretion is potentiated by Treg dysfunction, as well as reduced capacity of effector T cells to respond to Treg-derived cytokines. Cytotoxic CD4+CD28− cells also promote atherogenesis by inducing endothelial damage through various mechanisms. Finally, a potential role has emerged for other T cell lineages in autoimmunity-associated CVD; these include angiogenic T cells and CD1-restricted lipid responsive T cell subsets.

Because CVD is a major cause of morbidity and mortality in patients with systemic autoimmunity, targeting the immunologic drivers of vascular inflammation has the potential to substantially improve the quality of life of these individuals (78, 92, 113). Investigating the mechanisms of T-cell-mediated CVD in psoriasis has already culminated in late phase clinical trials, with additional studies ongoing (18, 20, 21). Ongoing investigations into the mechanisms by which T cell cells promote autoimmunity-related CVD will uncover additional therapeutic targets, allowing a more sophisticated approach to preventing and treating CVD in these cohorts. As systemic autoimmune diseases are present in up to 10% of the global population, these insights are likely to have a major public health impact (14). Ultimately, these findings may also have broader translational relevance to atherosclerotic CVD, where T cell dysfunction is also a major driver of vascular pathology.

## Author Contributions

DS, AB, MK, and YL: literature review, manuscript preparation, and generation of figures. DS and NM: oversight, editing, and planning. All authors contributed to the article and approved the submitted version.

## Funding

This project was funded by the ZIAAI001251; ZIAHL006193 and the National Psoriasis Foundation.

## Conflict of Interest

NM has served as a consultant for Amgen, Eli Lilly, and Leo Pharma, receiving grants and other payments. NH has served as a principal investigator and/or investigator for AbbVie, Celgene, Janssen Pharmaceuticals, Inc, and Novartis, receiving grants and/or research funding. NM has served as a principal investigator for the National Institute of Health, receiving grants and/or research funding.

The remaining authors declare that the research was conducted in the absence of any commercial or financial relationships that could be construed as a potential conflict of interest.
